# Planning pathways in the transfer of Directly Observed Treatment of Tuberculosis^1^

**DOI:** 10.1590/1518-8345.2213.3015

**Published:** 2018-08-09

**Authors:** Rarianne Carvalho Peruhype, Fernando Mitano, Juliana Feliciati Hoffmann, Catiucia de Andrade Surniche, Pedro Fredemir Palha

**Affiliations:** 2 Post-doctoral fellow, Escola de Enfermagem de Ribeirão Preto, Universidade de São Paulo, PAHO/WHO Collaborating Centre for Nursing Research Development, Ribeirão Preto, SP, Brazil. RN, Departamento de Ações em Saúde, Secretaria Estadual de Saúde do Rio Grande do Sul, Porto Alegre, RS, Brazil.; Secretaria Estadual da Saúde do Rio Grande do Sul, Departamento de Ações em Saúde, Secretaria Estadual de Saúde do Rio Grande do Sul, Porto Alegre, RS, Brazil; 3 PhD, Professor, Escola de Medicina, Universidade de Lúrio, Marrere, Nampula, Mozambique.; 4 Doctoral student, Faculdade de Medicina, Universidade Federal do Rio Grande do Sul, Porto Alegre, RS, Brazil. Statistician, Departamento de Planejamento Governamental, Secretaria de Planejamento, Governança e Gestão do Rio Grande do Sul, Porto Alegre, RS, Brazil.; Departamento de Planejamento Governamental, Secretaria de Planejamento, Governança e Gestão do Rio Grande do Sul, Porto Alegre, RS, Brazil; 5 Doctoral student, Faculdade de Medicina, Universidade Federal do Rio Grande do Sul, Porto Alegre, RS, Brazil. RN, Unidade de Terapia Intensiva Neonatal, Fundação Maternidade Sinhá Junqueira, Ribeirão Preto, SP, Brazil.; Unidade de Terapia Intensiva Neonatal, Fundação Maternidade Sinhá Junqueira, Ribeirão Preto, SP, Brazil; 6 PhD, Associate Professor, Escola de Enfermagem de Ribeirão Preto, Universidade de São Paulo, PAHO/WHO Collaborating Centre for Nursing Research Development, Ribeirão Preto, SP, Brazil.

**Keywords:** Health Planning, Tuberculosis, Public Policies, Primary Health Care, Public Health, Health Management

## Abstract

**Objective:**

to investigate the planning pathways in the transfer of Directly Observed Treatment of tuberculosis.

**Method:**

a qualitative study conducted using interviews and a semi-structured guide, administered to five subjects who were among the coordinators and managers of the tuberculosis control programs, and the secretary of health of a municipality in the south of Brazil. Situational Strategic Planning and Discourse Analysis of the French matrix were the theoretical and analytical references used, respectively.

**Results:**

three reflexive axes were identified: weaknesses in the process of planning the Directly Observed Treatment transfer, antagonism between planning and daily requirements and formulation of planning and execution. Lack of systematization regarding the planning and execution for transfer the Directly Observed Treatment policy, demonstrates the fragility and incipience of this activity, and the possibility of its non-existence.

**Conclusion:**

the urgent need for managers and coordinators to better appropriate the theoretical framework for changing public policies, and the related planning mechanisms, includes a proposal for reorganization and qualification of the diffusion process, both practical-operative and political-organization.

## Introduction

Discussing the importance of planning activity in the health area seems redundant and not very innovative. However, considering it in a context of public policy transfer constitutes an interesting challenge, mainly because it is an incipient and little-known topic in the Brazilian literature ^(^ ^1^.

We understand policy transfer as the process whereby “[...] knowledge about policies, administrative arrangements, institutions etc. in one time and/or place is used in the development of policies, administrative arrangements and institutions in another time and/or place”^2^. There are several interfaces and intercommunicating levels (global, national, regional, etc.) that permeate this knowledge, and several elements that need to be taken into account at the moment of its operationalization, such as the type of knowledge involved, the form of persuasion needed or those involved, the network of reference, the political, economic, social, and cultural context, and the resources employed, among others ^3^.

Although present in health, the scarcity of systematic studies on the process of public policy transfer in this field ^(^ ^4^ inspired us to investigate how planning the pathways in the transfer of directly observed treatment (DOT) policy for treating people with tuberculosis (TB) occurred in Porto Alegre, Rio Grande do Sul, which had one of the greatest incidence of this disease in 2016 (80.4 cases/100,000 inhabitants) among Brazilian capitals^5^.

The DOT can be understood as the observation of the patient’s daily intake of prescribed medication for the treatment of TB, from Monday to Friday, conducted by health professionals or anyone else trained or supervised by one of these professionals. However, for operational purposes, DOT can be considered as the follow-up of those patients with 24 supervised doses during phase one (loading dose), and 48 doses in phase two (maintenance)^6^. 

The correct use of medication enables a cure rate of 90% of TB cases^7^. Thus, DOT is an important strategy to ensure the patient intakes all the medications, promoting adherence to treatment and reducing the likelihood of possible medication resistance. In addition, scientific evidence shows that the difficulties of operationalizing DOT tend to significantly affect the achievement of improvements in cure rates and treatment abandonment^8^. 

However, low adherence and incorporation of this policy in primary care (PC) services are still found, along with a deficit by the municipal and state authorities regarding the linkage between demographic context, planning, and financing of programs designed to reduce costs generated by TB^9^.

Thus, the present study began with the following question: how has the planning for transferring the DOT policy been prepared, from the perspective of the TB program coordinators and managers in the different government levels (state and municipal)? The main study objective was to investigate the planning for transferring the DOT in the capital of the state of Rio Grande do Sul. For this purpose, the theoretical reference of Carlos Matus’ Situational Strategic Planning (SSP), and Pêcheuxtian Discourse Analysis (DA) were used as the theoretical-analytical references.

This study intends to be helpful as scientific evidence, as this remains a subject still little known in these domains, but especially for the reorganization of activities and services linked to the diffusion of DOT at different levels of government, as a reflexive and inductive instrument of process qualification, as complex as complementary, such as the planning and transfer of public policies.

## Methods

This was qualitative research, which used the Consolidated Criteria for Reporting Qualitative Research^10^, a checklist for the qualified description of the elements and steps taken in research of this nature.

The principal author conducted the interviews. An initial appointment with the subjects of the research was held by telephone and e-mail, during which information on the process, the day and dates for the development of this activity was provided. The research objectives and interest on the topic were explained during the initial contact for the interview.

In the present study, discourse analysis was adopted as the theoretical-methodological reference for recognizing the produced senses. French discourse analysis works with the constitutive crossing of three domains, namely: *linguistics* - does not focus on the language as an abstract system, but as a manner of signifying meaning; *Marxism,* with historical and dialectical materialism - the subject affected by history and by ideology; and, *psychoanalysis* - which deals with the subject of the unconscious^11^. 

The participants were intentionally selected, and included coordinators, managers of the TB control programs and the municipal health secretary of Porto Alegre, totaling five subjects. In addition to telephone contact and e-mail, they were addressed personally (face to face) at the time of the interview. All individuals agreed to participate in the study.

The interviews were held in the participants’ respective places of work and, although some of these locations were shared with other colleagues, this activity occurred in locations with the most privacy possible. The data collection occurred in 2014, using a semi-structured guide, which was evaluated by two specialists in DA, with the goal of improvement and qualification.

The final version with 10 questions included topics related to understanding the planning activity and its importance for healthcare, even to recognizing the operational stages and actions used in planning for the transfer of DOT, the agents and resources involved (financial, physical, organizational, etc.), among other elements.

The interviews, with an average length of 45 minutes, were audiotaped and later transcribed for analysis, along with a field diary prepared by the interviewer based on her personal perceptions. This diary provided additional information on the characteristics and functioning peculiarities of the place where the study was conducted, the work process, and the role played by the participants. There was no need to repeat the interview with the subjects.

Data were analyzed following the three steps of DA. The first, *from the linguistic surface to the discursive object*, consisted of repeated readings of the transcribed interviews, analyzing the discourse, and trying to identify indicators for interpretation, and denaturalizing the word of things^11^. 

The second step, *from the discursive object to the discursive process*, consisted of identifying discursive sequences (DSs) and relating them to the different discursive formations that delineate the circulating senses in the fragments under analysis. Finally, the third step, the *discursive process* *itself* (*ideological formation*), consisted of the interpretation of discursive sequences, considering the existing conditions of production and the discursive formations in which the statements were anchored^11^ as well as the mobilization of the subjects, including the French DA and the subject under study (transfer of public policy and planning), to support the interpretative arguments.

Among the five subjects interviewed, three DSs were selected for analysis because they were ideologically representative of the subject-positions assumed by the interviewees. It should be noted that, in DA, vertical exhaustiveness is the most relevant, in-depth exploration of the empirical object, to the detriment of horizontal saturation, which evidences completeness through its extension^11^. Consequently, the *corpus* of the research is not necessarily related to the number of selected DSs, and can consist of a single DS, as long as the established objective is met. Thus, three main reflexive axes derived from the data analysis were found, namely: *weaknesses in the process of planning the DOT transfer*, *the antagonism between planning and daily requirements,* and *formulation of planning and execution*.

The research project was submitted to and approved by three research ethics committees, and included only subjects who agreed to participate by signing the Terms of Free and Informed Consent form, having at least six months of experience with DOT. Those on vacation or health leaves during the data collection period were excluded.

The limitations of the present study are those related to elements present in the work environment, identified during the interviews (interruptions by colleagues, telephone, overwork, pressed for time to accomplish tasks, as well as the spatial arrangement of tables and seats, typical of shared spaces), which might generate some type of inhibition and influence the participants’ responses.

## Results and Discussion

In the reflective axis, weaknesses in the process of planning the DOT transfer, a DS is stated in which the subject, when asked about the planning process for transfer of this policy during its coordination, responded as follows: *This is what we saw in 2008; that one of the problems is related to the program, an adherence issue. And then we begun to discuss: what can we do to ensure that patients can get through the end of treatment, or not abandon it in the first and second months of treatment?* *And, of course, if you look into the literature, you will find publications, and the World Health Organization itself is talking about DOT for many years, right? Then, we initiated a discussion, with the teams and with the managers of the service, on the possibility of DOT. And, in 2009 the Global Fund was working and several discussions encouraged the implementation of the Directly Observed Treatment, and then, I said: it’s good for us, to get a resource, which we never get, to train everyone* [...] (Subject 1).

From the fragment, we realized that the subject initially began from a problem (explanatory moment of SSP) with the scope of the operationalization of the policy, which is the patient’s adherence to treatment, to indirectly identify the problem related to the DOT policy transfer: the lack of resources for staff training and qualification. Low adherence and the need for reversing the situation, the international theoretical contribution of the World Health Organization to the DOT and the incentive, including financial support from the Global Fund seems to be part of the problems and opportunities that drive the planning and, the implementation of the DOT transfer.

Experiences in Mozambique have demonstrated that the political process of transferring the DOT short-course of TB control strategy, in which a supervised treatment is one of the components, has also been significantly influenced by scientific, technical data, as well as transnational financial resources^12^. 

However, although there are indications of a starting point (problem) and of the agents involved in the process, as will be discussed later, lack of clarity and details regarding the planning step of the DOT transfer were identified. The systematization and degree of formalization of the process remains very incipient and shallow. If the management of the Unified Health System (the Brazilian Sistema Único de Saúde -SUS) is considered out of date, with deficits in service planning and evaluation^13^, this naturally reflects upon the scope of other proposals, especially those that are still little known and exploited, such as the transfer of a public policy.

In the statement *we began to discuss with the teams the possibility of* DOT, clues indicate that the agents involved in the process (coordinators and health team) were found. However, it remains impossible to determine if the content of this discussion incorporated aspects other than the possibility of policy operationalization. It seems that the significant discussion referred more to the implementation of DOT (discuss the possibility of DOT, several discussions encouraging the implementation of Directly Observed Treatment) than to planning the moment for transferring the policy itself.

The feasibility of training, as a possible element for the transferal process, seems directly correlated to the existence of resources, which is aggravated by the fact that TB still appears as a neglected disease, without the structural and organizational quality necessary for control and treatment in many health services^14^. In fact, the development of human resources for TB control was so important that it was considered as an element of a strategic plan, in 2006-2010, for Africa, America, Southeast Asia and Western Pacific regions^15^.

The results of a study conducted in Divinópolis, Minas Gerais, demonstrated that the local TB control program had as difficulties: a fragile planning process, lack of a standard model for the diffusion of information and data, lack of a training program and trained professionals, who in turn, demonstrated lack of knowledge in diverse areas such as surveillance, TB diagnosis, and accomplishing the DOT^16^. In Cabedelo, Paraíba, other factors emerged as barriers in the management and performance of primary care services in relation to TB treatment, such as: the fragmentation of professional practice, lack of systematization in home care, and focus of the professional qualification^17^ .

Hesitations represented by the murmurs, *hum* and *well*, cannot specify what actually motivated the subject. The problems and objectives no longer have fixed seats and the substantive *opportunity* becomes polysemic: to obtain financial resources, to promote the qualification of professionals, and to improve patient compliance and DOT implementation. Nothing, or very little about the plan for policy transfer, was observed.

Thus, the marked lack of systematization of planning on execution of the DOT transferal demonstrates not only the fragility of this activity, but also the possibility of its non-existence. Commonly, planned actions are not always performed, and routinely demand as “putting out fires”, that not only distort and make the practice of planning unfeasible, but, above all, reinforce a less thoughtful and well-founded management practice^18^.

The importance of planning for TB control is scientifically proven. The National Tuberculosis Control Program in Brazil, seeks to improve the qualification of processes such as planning, monitoring, evaluation of control actions, prevention, care, diagnosis, epidemiological surveillance, etc.^16^ A study conducted in the Tak province, Thailand, demonstrated that planning, was one of the main elements for disease control in that setting, along with effective sharing of data and information, and improvement in the diagnosis process and care, among others things^19^.

Regarding the axis of *the antagonism between planning and daily requirements*, the response of another subject when asked about the periodicity of review, re-evaluation, and reformulation of planning for the transfer policy of DOT was: *Look [laughs], unfortunately, periodicity as we would like does not exist; it is faced as when we found the emergence room crowded, the isolation full of patients, many cases of tuberculosis, then, the people* [...] *for me is a phone call like this: ah! You are very experienced, you know the workflow, you know the network, come in and help us because we are worried about tuberculosis, then it starts over again: there we* [...] *but we have a plan, look here, just apply it. Ah! So let’s see what we do first [laughs], and so on* (Subject 2).

The uneasiness that erupts at the beginning of this DS - *Look* [laughs] - enables us to glimpse the possibilities of conflict and contradiction of the subject. When we consider the sentence, “*look* [laughs], *unfortunately, periodicity as we would like does not exist”,* we perceive the tenuous borders of ambivalence mainly in planning and in the periodicity of its evaluation.

The tension, in this case, lies in the semantic struggle of *we* *would like* and *unfortunately*, between what the subject wants and possesses, the ideal and the real, between the positive and the negative, that is, multiple intonations in a voice captured ideologically, which become individual as well as collective, singular and plural. Although planning is considered to be an important activity, the maintenance of the current system still overlaps the managers’ rating scale. They, in turn, need to plan actions that consider the real needs of the population, with proposals for change that consolidate the various health services offered^20^.

The tension of this sentence extends to the practical operational field of planning, in which *unfortunately* incorporates the limitation of the activity itself and, perhaps, even the passivity of the subject for reverting the situation. We know the importance of a periodic evaluation of a plan, which is a unique opportunity to qualify the process, to guide the decision-making, and to adapt the resources to achieve the goals. As observed, there is still a long distance between wanting (*we would like*) and doing it (*unfortunately*).

In this DS, we again noticed that the planning that the subject mentions seems to be more related to the operationalization of the TB control policy than to the transfer of the TB policy, along with the aggravation of still being used for emergency purposes and perhaps of improvisation - *it is faced as when the emergency room is crowded, the isolation is full of patients, many cases of tuberculosis* [...], reinforcing the idea that the absence of articulation and planning in health services can lead to a vicious circle based on caring for the spontaneous demands to the detriment of the programmed actions^21^.

The expression*, then it starts over again*, installs a new semantic conflict in the subject discourse, as it can not specify what is restarted, whether it is the mobilization of a supposed plan to face everyday emergency situations, or the awareness of other individuals about the existence of a plan, or both. The continuity of action and the cyclical character, suggested in the subject’s discourse, lead us to think about the myth of Sisyphus. According to Greek mythology, Sisyphus received, as a punishment from Zeus, the task of pushing a heavy stone to the top of a mountain, from which it always rolled back to the bottom, requiring a continuous resumption of his effort^22^.

However, when stating, “*but we have a plan, look here, just apply it”*, we notice an important point: whatever the established purpose of this plan is, a certain lack of knowledge by other people directly linked to its operationalization remains. If the participation of other social agents in planning is one of the strengths of the SSP^23^, in this context it seems to be playing a secondary role. Such ignorance can be ratified by the surprised intonation of these *others* - “*Ah*!” - and by the search for understanding and prioritizing actions: “*So let’s see what we do first.”*

Whether the uneasiness introduces this DS, it also ends it “[*laughs*] *and so on*,” leaving us indications of an uncertain cycle of continuity and configuration: we do not know what is perpetuated: whether is the lack of periodicity in assessment of the plan, or the repeated activation of the subject by the other agents of the process, or the repeated application of this plan, which the other subjects seem not to know, in every day and emergency situations, or even all of them. What we do know, however, is that the potential for articulation and development of interdisciplinary team work is directly influenced by the existence and guarantee of spaces for discussion, whose absence, in turn, can culminate in individualized planning, in which each professional defines and programs his/her actions, with disarticulation of the potential of collective action^24^.

Regarding the *formulation of planning and execution* axis, when Subject 3 was asked about the strategies and the financial, human, physical and organizational resources used to formulate and execute the plan for the DOT transfer process, the answer was (DS extracted in two different moments - M1 and M2 - of the same answer): M1 *- “Hmm, not like us, not in financial terms, to be able to execute, we are basically running at zero cost. Everything we were able to acquire was to increase of the transit passes for transportation of professionals to execute the DOT, or possibly some patient to the health unit when this is necessary”* [...]*.* M2 *- “material, we are using copies, we have not been able to print any specific material for this training so far, so the cost is basically to provide food for those completing the training. The entire planning is done on a territorial basis between the local management center and the territorial reference center* (Subject 3)*.*

In M1, this subject, as well as the others, seems to address elements aimed at the DOT performance - acquisition of “*transit passes for transportation of professionals to execute the DOT, or possibly some patient”*, which may have been due to the lack of knowledge on the subject of the transfer of public policies, among other things. A similar result was obtained in a study conducted in João Pessoa, Paraíba, in which the managers showed a lack of knowledge about this subject, and also in a context of DOT transfer, culminating in a significant weakening in the development of TB control actions^9^ .

However, in M2, we observe a redirection of the statement, with incorporation of elements allusive to the process of transference of policies, such as, for example, the mention of the term *training* again. Certainly, the health professional needs to be able to take a dialogical approach and to empower the patient with TB, disseminating knowledge and promoting awareness about the treatment of the disease. However, the degree of preparation of professionals in this area is still nothing like what is desired, which requires the implementation of a policy of continuing education in the health services^25^ and, consequently, a qualification of the policy transfer process.

In both moments, M1 and M2, there were also no indications that referred to the formulation of the planning of the DOT transfer. In M1, the execution of the policy seems not having been preceded by adequate planning, with an emphasis on the analysis of financial “viability” that is fundamental for this type of activity: *we are basically running at zero cost*.

In the M2, we cannot specify on which territorial basis the planning was performed, or is yet to be performed, between the local management and the territorial reference center. Semantically, however, we observe the possibility of a collective development and a living, dynamic, non-static territory that ascends to the idea of ​​trainings, and of the very elaboration of a plan. We cannot lose sight of the fact that the success in implementation of public policies occurs by institutional structures enabling negotiations and debates among the multiplicity of agents in the respective decision-making process, among other factors^26^.

If planning, in turn, is considered to be an indispensable tool at any level of operationalization of actions, proposals, services, strategies, and policies in health and other areas, it seems to us that once again it was turned down by the subject of the statement related to the transfer of DOT. The indefinite pronoun *everything* and the adverb *basically*, for example, suggest complements that limit and link the action of the subject to the instance of the policy execution (acquisition of *transit passes for transportation* and *food*, respectively), without, however, showing some clues of the planned method for the policy transfer.

Thus, considering that a strategic action implies planning as a form of viability development^27^, a point also emphasized in the SSP of Matus^23^, we must believe that the absence or incipience of this activity can have serious consequences for reaching the goals. A poor qualification in the formulation, or the very lack of planning of the policy transfer, can result in a nonsystematized process of proposal diffusion that extends from one level to another in the management, with a possibly negative impact not only for those who operate it, but also especially for those who need it.

This can be an important shortcoming in the management and organization of processes related to DOT and TB control in the capital of Rio Grande do Sul, whether related to planning or to the transfer and execution of the policy, contributing, decisively, to the lack of goal achievement and preventing the desired improvement in health indicators of the population. 

## Conclusion

If the main objective is to qualify the execution of policies and programs by health professionals, there is a clear and urgent need for managers and coordinators to better appropriate both the theoretical framework of the policy transfer process, and the planning mechanisms themselves. 

The results obtained in this study enable us to discuss the need for a reorganization of the activities (planning/transfer) related to the DOT, which permeate the appropriate understanding of the various stages and elements contained in these different processes. This means an adequate mobilization of the agents involved, a better understanding of what to do in political time and in the territorial context, the financial and operational feasibility of the proposal, and the importance of standardization and systematization of actions and instruments. The fact is that it is not enough to have theoretically perfect public policies, which become ineffective due to the lack of qualification of the diffusion process, practical-operative and political-organizational.

As it is still a serious public health problem, an even more cautious look at the process of transferring policies aimed at controlling TB is required, and the planning of this stage is a operational foundation. Such an understanding is inclusive and should be broadened to other sectors and health policies at the diverse government levels, which qualifies the present experience as the first one of many others that may follow

## References

[1] Farah MFS (2008). Disseminação de inovações e políticas públicas e espaço
local. O&S.

[2] Dolowitz D, Marsh D (1996). Who learns what from whom a review of the Policy Transfer
literature. Polit Stud.

[3] Bissell K, Lee K, Freeman R (2011). Analysing policy transfer perspectives for operational
research. Int J Tuberc Lung Dis.

[4] Freeman R (1999). Policy transfer in the health sector. European Forum Conference paper
WS/35.

[5] Ministério da Saúde (BR). Secretaria de Vigilância em Saúde (2017). Indicadores prioritários para o monitoramento do Plano Nacional
pelo Fim da Tuberculose como Problema de Saúde Pública no
Brasil. Bol Epidemiol.

[6] Ministério da Saúde (2011). Manual de Recomendações para o controle da Tuberculose no
Brasil.

[7] Stop TB Partnership (2015). The Global Plan to stop TB 2011-2015: transforming the fight towards
elimination of tuberculosis.

[8] Souza KMJ, Sá LD, Silva LMC, Palha PF (2014). Nursing performance in the policy transfer of Directly Observed
Treatment of Tuberculosis. Rev Esc Enferm USP.

[9] Oliveira RCC, Adário KDO, Sá LD, Videres ARN, Souza SAF, Pinheiro PGOD (2016). Managers' discourse about information and knowledge related to
Directly Observed Treatment of Tuberculosis. Texto Contexto Enferm.

[10] Tong A, Sainsbury P, Craig J (2007). Consolidated criteria for reporting qualitative research (COREQ)
a 32-item checklist for interviews and focus groups. Int J Qual Health Care.

[11] Orlandi EP (2012). Análise de discurso: princípios e procedimentos..

[12] Cliff J, Walt G, Nhatave I (2004). What's in a Name Policy transfer in Mozambique: DOTS for
tuberculosis and Syndromic Management for Sexually Transmitted
Infections. J Public Health Pol.

[13] Lorenzetti J, Lanzoni GMM, Assuiti LFC, Pires DEP, Ramos FRS (2014). Health management in Brazil dialogue with public and private
managers. Texto Contexto Enferm.

[14] Protti ST, Silva LMC, Palha PF, Villa TCS, Ruffino-Neto A, Nogueira JA (2010). Managing the basic health unit in tuberculosis control: a field
of challenges. Rev Esc Enferm USP.

[15] Oliveira LGD, Natal S, Chrispim PPM (2010). Directly Observed Treatment: strategy to Tuberculosis
control. Rev APS.

[16] Andrade HS, Oliveira VC, Gontijo TL, Pessôa MTC, Guimarães EAA (2017). Evaluation of Tuberculosis Control Program a case
report. Saúde Debate.

[17] Silva DM, Nogueira JA, Sá LD, Wysocki AD, Scatena LM, Villa TCS (2014). Perfomance evaluation of Primary Care services for the treatment
of Tuberculosis. Rev Esc Enferm USP.

[18] Silva BFS, Wandekoken KD, Dalbello-Araujo M, Benito GAV (2015). The relevance of planning as management practice in health
microregion of São Matheus (ES). Saúde Debate.

[19] Tschirhart N, Thi SS, Swe LL, Nosten F, Foster AM (2017). Treating de invisible Gaps and opportunities for enhanced TB
control along the Thailand-Myanmar border. BMC Health Serv Res.

[20] Passos RA, Nunes SS, Silva LF (2015). The plurality of the concept of health: the voice power of
Unified Health System (SUS) users in a municipal health
conference. Rev Cienc Saúde.

[21] Peres AM, Freitas LJ, Calixto RC, Martinez Riera JR, Sanjuan Quiles A (2013). Conceptions of nurses regarding planning, organization and
management of nursing in primary care: integrative review. Rev Enferm Ref.

[22] Rocha NMFD, Góis CWL (2010). Trajectories of young people in the world of work from the first
insertion the case of Sisyphus Maracanaú - in Ceará, Brazil. Psicol Soc.

[23] Matus C (1991). O plano como aposta. São Paulo Perspect.

[24] Bazzo-Romagnolli AP, Gimenez-Carvalho B, Almeida-Nunes EFP (2014). Management of the Basic Health Unit in small municipalities
instruments, facilities and related difficulties. Rev Gerenc Polit Salud.

[25] Cecilio HPM, Marcon SS (2016). Health personnel's views of directly observed treatment of
tuberculosis. Rev Enferm UERJ.

[26] Loureiro MR, Abrucio FL (2012). Democracia e eficiência a difícil relação entre política e
economia no debate contemporâneo. Rev Econ Polit.

[27] Rodrigues AL, Sarriera JC (2015). Training of managers in a Community Violence Prevention Program
Fractal. Rev Psicol.

